# Generation of a Single Chain Antibody Variable Fragment (scFv) to Sense Selectively RhoB Activation

**DOI:** 10.1371/journal.pone.0111034

**Published:** 2014-11-03

**Authors:** Patrick Chinestra, Aurélien Olichon, Claire Medale-Giamarchi, Isabelle Lajoie-Mazenc, Rémi Gence, Cyril Inard, Laetitia Ligat, Jean-Charles Faye, Gilles Favre

**Affiliations:** 1 Inserm, UMR 1037-CRCT, GTPases Rho dans la progression tumorale, Toulouse, France; 2 Université Toulouse III-Paul Sabatier, Faculté des Sciences Pharmaceutiques, Toulouse, France; 3 Institut Claudius Regaud, Toulouse, France; 4 CRCT, plateau de protéomique, Toulouse, France; National Cancer Institute, NIH, United States of America

## Abstract

Determining the cellular level of activated form of RhoGTPases is of key importance to understand their regulatory functions in cell physiopathology. We previously reported scFvC1, that selectively bind to the GTP-bound form of RhoA, RhoB and RhoC. In this present study we generate, by molecular evolution, a new phage library to isolate scFvs displaying high affinity and selectivity to RhoA and RhoB. Using phage display affinity maturation against the GTP-locked mutant RhoAL63, we isolated scFvs against RhoA active conformation that display K_d_ values at the nanomolar range, which corresponded to an increase of affinity of three orders of magnitude compared to scFvC1. Although a majority of these evolved scFvs remained selective towards the active conformation of RhoA, RhoB and RhoC, we identified some scFvs that bind to RhoA and RhoC but not to RhoB activated form. Alternatively, we performed a substractive panning towards RhoB, and isolated the scFvE3 exhibiting a 10 times higher affinity for RhoB than RhoA activated forms. We showed the peculiar ability of scFvE3 to detect RhoB but not RhoA GTP-bound form in cell extracts overexpressing Guanine nucleotide Exchange Factor XPLN as well as in EGF stimulated HeLa cells. Our results demonstrated the ability of scFvs to distinguish RhoB from RhoA GTP-bound form and provide new selective tools to analyze the cell biology of RhoB GTPase regulation.

## Introduction

The members of the large family of monomeric GTP-binding proteins, or small G proteins, function as molecular switches triggering signalling cascades involved in the regulation of a wide variety of cell processing. They serve as key regulators of extracellular-stimuli-transducers that mainly direct actin reorganisation, cell-cycle progression and gene expression [1] and have been implicated in cancer progression [2]. Monomeric GTPases cycle between an inactive GDP-bound to an active GTP-bound state that differ by the positioning of the switch I and switch II domains [3]. The active conformation interacts with effector proteins to induce downstream signalling events. Guanine nucleotide Exchange Factors (GEFs), promoting the release of bound GDP and its replacement by GTP, activate the Rho GTPases. GTPase-activating proteins (GAPs) accelerate the GTP hydrolysis and turn off the RhoGTPase to the inactivated GDP-bound form. RhoGTPases are anchored to membranes by prenylated carboxy terminal cysteine and are also regulated by Guanine nucleotide Dissociation Inhibitors (GDIs), which main known function is to maintain GTPases in soluble inactive complexes [4]. The Ras superfamily is structurally classified into seven families: Ras, Rho, Rab, Sar1/Arf, Ran, MIRO and RhoBTB3 [5]. Rho proteins comprise 20 members that differ from other GTPases by the presence of an insert loop. Among the Rho proteins we focus on RhoA, RhoB and RhoC, which have long been confused in their biological activities because of their high amino acid sequence homology. Indeed, RhoB shares more than 80% homology [6] with RhoA and RhoC while RhoA and RhoC identity reaches 92%. However, it is now admitted that they differ in many biochemical characteristics and cellular functions. RhoA and RhoC are constituvely expressed while RhoB is an early inducible gene. RhoA and RhoC localize to the plasma membrane while RhoB has been found associated both to the plasma membrane and to the endosome [7], [8] and more recently acting at the nuclear level [9]. Lastly, we [10] and others [11] have demonstrated that RhoB but not RhoA or RhoC displays gene suppressor activity in many cancer types and is critical to control cell survival upon genotoxic stress [12], [13] or even in DNA damage response [14].

To date, the reference tool to evaluate the GTP-bound form of Rho in cell extracts is based on a pulldown assay relying on the Rho binding domain of rhotekin (RBD) as the bait [15]. One main caveat of this approach is that the RBD effector domain lacks of selectivity towards the three activated forms of RhoA, RhoB and RhoC homologues, and have low affinity to the Rho proteins. Another limitation resides in the poor stability of the RBD recombinant polypeptide which require to be purified only as a GST-fusion. There is a real need for reliable and selective tools, more versatile to investigate the cellular activation of RhoGTPases.

The detection of the level of single activated Rho is still challenging and would represent a significant progress in the study of their biological role. In this vein of research, we have previously reported the characterization of the scFvC1 conformational sensor selective of RhoA, RhoB and RhoC activated forms [16] but with a relative low affinity (K_d_ = 3 µM). We achieved a new scFvs library through molecular evolution of scFvC1 and performed affinity maturation selections with phage display technology. We obtained several scFvs exhibiting a strong improvement of affinity reaching the nanomolar range. Furthermore a substractive selection strategy led to the identification of scFvs discriminating RhoB from RhoA in their active conformation, despite a near 100% identity in the switch I and switch II domains [3]. Moreover, we demonstrated that these scFv selectively recognize cellular activated form of RhoB providing new tools to study RhoB functions.

## Materials and Methods

### Construction of a mutant library for scFv C1 antibody affinity maturation and cloning

The starting material for library construction was the pHEN C1 phagemid previously described [16]. Plasmid DNA was submitted to random mutagenesis by epPCR using GeneMorph II EZClone Domain Mutagenesis Kit (Stratagene) according to the high mutation rate protocol using the C1 specific upstream primer 5′TTATTACTCGCGGCCCAGCCGG3′ that hybridized just upstream of the NcoI restriction site of the pHEN vector and downstream primer 5′ GTGATGGTGATGATGATGTGC 3′. The mutated gene fragments were gel purified and digested with NcoI and NotI (New England Biolabs) followed by ligation into the corresponding sites of the pHEN phagemid containing an irrelevant scFv in order to avoid the presence of the native scFvC1 in the subsequent steps of affinity maturation. The library was subsequently transformed into electro competent XL1 blue *Escherichia coli* (*E.coli*) (Stratagene) and random clones sequenced with the primers LMB3 5′ACAGGAAACAGCTATGACC3′ and pHEN-SEQ 5′CTATGCGGCCCCATTCAG3′.

### Affinity maturation, phage display selection and screening

Phage stocks of the library, phage display selection and screening techniques were performed as previously described in detail [17]. Briefly, we used mutant L63 of Rho (locked in GTP binding structure) expressed in BL21 *E.coli* strain as a GST-fusion protein captured in glutathione coated micro wells (Pierce). After blocking the well with PBS containing 3% non-fat dried milk powder (MPBS), phages (10^9^ TU) were added and allowed to incubate for 30 min under stirring followed by 90 min without shaking at room temperature. After extensive PBS/0.1% Tween-20 and PBS washes used to minimise non-specific phage interaction, phages were eluted by adding triethylamine (1.4% in water) and subsequently neutralized by 1 volume of Tris-HCl (1 M, pH 7). Eluted phages were amplified by infecting exponentially growing XL1 blue *E.coli* and subsequently plated on ampicillin containing agar plates for CFU titer determination and further analysis by colony PCR and DNA sequencing.

Affinity maturation experiments were performed as described above by varying the washing stringency and by lowering the concentration of antigen. Briefly, for the first two rounds of selection, phages were incubated with saturating concentration of GST-RhoAL63-bound glutathione coated micro well (1 µg). In the first round, wells were washed 10 times with PBS/0.1% Tween-20 followed by 3 times with PBS while the second one consisted in 20 washes with PBS/0.1% Tween-20 followed by 5 washes with PBS. The subsequent two rounds were performed with decreasing concentrations of antigen determined by ELISA and corresponding to the concentration of antigen giving 70% and 30% of the maximal binding of phages from the previous round respectively. Consequently we coated wells with 50 ng/mL of antigen for the 3^rd^ round and 5 ng/mL for the 4^th^ round.

For the isolation of scFv against RhoB active conformation, the first round of selection consisted in incubating phages with 0.5 µg of the mutant GST-RhoBL63 bound to glutathione coated micro wells. The subsequent two rounds were conducted with a subtractive selection procedure consisting in a preincubation of phages with soluble GST-RhoAL63 (10 µg) during 1 h followed by another one hour in the presence of glutathione coated beads in order to eliminate RhoAL63 bound phages. After a brief centrifugation the remaining unbound phages were incubated with 0.5 µg of the mutant GST-RhoBL63 bound glutathione coated micro wells, washed and eluted as described above.

Affinity maturation and selection of conformation-specific scFv against the active form of RhoB were monitored by polyclonal phage ELISA on captured GST-Rho as previously described [17].

### Antigen preparation for scFvs library selections

Recombinant GST fusion Rho proteins were expressed and purified in a protease deficient strain (*E. coli* BL21) as previously described [17].

The cDNAs encoding RhoAL63, RhoBL63 or RhoA wild type were inserted into the pHIS parallel2 vector from Dr. P. Sheffield [18], in-frame at the 3′ end of the 6xHIS tag. Recombinant proteins were expressed in a protease deficient strain (*E. coli* BL21) and were subsequently purified under native condition using Ni-NTA resins (QIAGEN) according to manufacturer's instructions. Purified recombinant proteins were resolved on a 12.5% SDS-PAGE gel and visualized by Coomassie staining. Measuring absorbance at 280 nm assessed protein concentrations.

### Production and purification of single-chain Fv antibody

Soluble scFvs from selected clones were subcloned in fusion with the N-terminal domain of the phage P3 (NP3) protein necessary for their binding capabilities when expressed as soluble fragments as previously described for the scFvC1 [16]. Fusion scFvs were expressed in XL1 blue *E.coli* and were purified from periplasmic fraction using Ni-NTA resins (QIAGEN) as previously described [17]. Purified scFvs were then concentrated with the ProteoSpin CBED Micro Kit (Norgen) according to the protocol for acidic proteins. scFvs were resolved on a 10% SDS-PAGE gel and visualized by Coomassie staining. Measuring absorbance at 280 nm assessed protein concentrations.

For CBD pull down experiments, scFvs were expressed in fusion with the Chitin Binding Domain tag (CBD). The NP3 fragment from pHEN scFv-NP3 and the CBD fragment from the pTYB1 plasmid (New England Biolabs) were PCR amplified in order to introduce a BglII site at the 3′ and 5′ fragments extremities respectively with the following primers: P3-CBD-s 5′GTGCGGCCGCACATCATCATCACC3′, P3-CBD-as 5′TAGATCAGATCTGGATCCACGC- GGAACCAGAGAGCCGCCGCCAGCATTGACAGG3′ and CBD-Bgl-s 5′TAGCTAAGATCTGG- GATTACTTTATCTGATGATTCTGATC3′, CBD-Eco-as 5′TCAGTAGAATTCTTATCATTGAA-GCTGCCACAAGGCAGG3′. The NP3 PCR fragment was digested NotI/BglII and the CBD PCR product by BglII/EcoRI. The NP3 fragment of the pHEN-scFv-NP3 was removed by NotI/EcoRI digestion and replaced with the two PCR amplified fragments in a trimolecular ligation in order to obtain the pHEN-scFv-NCBD plasmid.

scFv-NP3-CBD clones were expressed in XL1 blue *E.coli* as described above. Culture supernatants added to periplasmic extracts were incubated with chitin beads (New England Biolabs) by means of a peristaltic pump at 4°C according to the manufacturer's instructions. scFvs bound to chitin beads were stored at −80°C in TBS/50% glycerol until use.

### ELISA experiments

For the screening of selected clones and the assessment of specificity, ELISA were performed as previously described [17]. Recombinant GST-Rho proteins were incubated on Reacti-Bind glutathione coated plates for 1 hour at room temperature. Phages or purified scFvs were revealed with antibodies anti-M13-HRP (GE Healthcare) or anti-c-myc-HRP (Novus Biologicals) respectively. Nucleotide loading of recombinant Rho from crude bacterial extracts was performed as previously described [17].

A method described by Friguet [19] was performed to determine K_d_ values. While coating GST-Rho on Reacti-Bind plates, purified scFvs (10^−9^–10^−8^ M) were incubated until equilibrium (30 min, 23–25°C) with a range of concentrations of soluble 6xHis-RhoAL63 or 6xHis-RhoBL63. The antigen-scFvs complexes were then transferred to the GST-Rho-coated plates and incubated for 10 min (23–25°C). After washings, the free scFvs fraction was quantified using antibody anti-c-myc-HRP. Before performing these assays, parameters such as time of equilibrium, linearity of the curve and time of incubation for the quantification of the free scFvs fractions were determined as previously described by Martineau [20]. K_d_ values were determined by non-linear regression of the curves plotting the ratio A_0_-A/A_0_ against the range of concentrations of soluble antigens using Prism (Graphpad) software, where A_0_ and A are the absorbance in absence and in presence of soluble antigens, respectively.

### Surface Plasmon Resonance assays

All binding studies based on SPR technology were performed on BIAcore T200 optical biosensor instrument (GE Healthcare). Immobilization of anti-GST antibody (30 µg/ml) to capture GST fusion proteins (GST-RhoAL63, GST-RhoBL63 and GST-RhoC) was performed by amine coupling on Sensor Chip CM5 in HBS-P buffer (10 mM Hepes pH 7.4, 150 mM NaCl, 0.005% surfactant P20) (GE Healthcare). Capture of Rho-GST proteins was performed at a flow rate of 10 µl/min with a final Protein concentration of 10 µg/ml. Total amount of immobilized anti-GST antibody was 7500 RU and total amounts of captured GST fusion proteins were about 1000 RU. GST alone was captured on channel (Fc1) for non-specific binding measurements. Fc1 was used as a reference channel. Binding analyses were performed with antibodies at different concentrations over the immobilized GST fusion protein surface at 25°C for 2 minutes at a flow rate of 30 µl/min. A single-cycle kinetics (SCK) analysis to determine association, dissociation and affinity constants (ka, kd, and K_d_ respectively) was carried out by injecting different antibodies concentrations (100 nM–6.25 nM). Binding parameters were obtained by fitting the overlaid sensorgrams with the 1∶1 Langmuir binding model of the BIAevaluation software version 2.0.

### GST and CBD pull down experiments

HeLa S3 cells ((Cervical adenocarcinoma; ATCC, CCL-2.2) (3×10^6^ cells) were seeded on to 145 cm^2^ tissue-culture dishes and grown in Dulbecco's modified Eagle's medium (DMEM) (Cambrex Lonza) supplemented with 10% fetal calf serum. For nucleotide loading, cells extracts (see below) were incubated with 1 mM GDP or 100 µM GTPγS, 10 mM EDTA at 30°C for 45 min and supplemented with 60 mM MgCl2. For XPLN overexpression, cells were transfected with pRK5-XPLN (generously provided by Alan Hall) or pEGFP (Clontech) as a control of transfection using JetPRIME (Polyplus) method. For EGF stimulation experiments, HeLa cells were cultured in serum free media for 24 h before addition of EGF (Sigma) (2.5 ng/mL) for 10 min. Cells were scraped in 400 µL ice cold lysis buffer (50 mM Tris–HCl, pH 7.5, 500 mM NaCl, 10 mM MgCl2, 1% (v/v) Triton X-100, proteases inhibitors (Sigma-Aldrich)) mixed thoroughly and cleared by centrifugation at 16 000xg for 2 min at 4°C. An aliquot (5 or 10%) from each lysate was taken as input controls. Analysis of the level of activated Rho were performed by using the method initially described by Ren et al. [15] that is the GST fusion protein containing the Rho binding domain of the downstream effector rhotekin and adapted to RhoB [21]. Briefly, the Rho binding domain of rhotekin (RBD), an effector of Rho proteins that selectively binds to the GTP-loaded form, was expressed as a recombinant fusion with GST in *E.coli* and purified through binding to GST-Sepharose beads. Cells treated extracts were incubated with either 10–20 µg of GST-RBD or 0,5–1 µg of scFv-bound chitin beads and rotated for 45 min at 4°C. Beads were washed three times with ice-cold wash buffer (50 mM Tris-HCl, pH 7.5, 150 mM NaCl, 10 mM MgCl2, 1% (v/v) Triton X-100). Bound proteins were eluted from the beads with SDS-PAGE sample buffer at 95°C and separated on 12.5% SDS-PAGE for Western Blot analysis with anti-RhoA (Cell Signaling Technology) or anti-RhoB (Santa Cruz Biotechnology) antibodies followed by HRP-conjugated secondary antibodies (Bio-Rad). Visualization of proteins on Western blots was performed either with the ECL Western blotting substrate detection system (Pierce) or the ChemiDoc imaging system (BioRad).

### Molecular modeling

All structures information were retrieved from the RCSB Protein Data Bank (www.rcsb.org): The PDB structure entry for active RhoAV14-GTPγS is 1A2B [3]. The PDB ID for Mg-Free form of RhoA-GDP is 1DPF [22] and 2FV8 for Mg-Free form of RhoB-GDP [23].

Molecular surface of RhoA and structural superposition of RhoA & RhoB were performed by the *UCSF-Chimera* Package (http://www.cgl.ucsf.edu/chimera) [47]: The molecular surface was created by the *MS/MS surface tool* with the parameter “Probe radius” set to 1.4 Å. The *Matchmaker tool* was used to generate the structural superposition with the following settings (Alignment algorithm: Needleman-Wunsh; Matrix: BLOSUM-62).

## Results

### Library construction and affinity maturation

The aim of our study was to generate scFv antibodies selective towards the active form of the small GTPases RhoA or RhoB. Our strategy is based on the improvement in terms of binding affinity and selectivity, through molecular evolution, of the previously described scFvC1 [16]. We demonstrated its ability to bind selectively to the active form of RhoA, RhoB and RhoC. To perform a molecular evolution of the scFvC1, diversity was introduced randomly by error-prone PCR (epPCR) into its coding DNA as previously described [24]. Mutation rate protocols of the GeneMorph II EZClone Domain Mutagenesis Kit (Stratagene), described in experimental procedures permitted us to obtain a library having a random nucleotide substitution frequency of 0.24%. This corresponded to a range of 1-5 amino acid substitutions taking into account that 20% of the clones sequenced did not exhibit any mutation. This frequency of mutation is located in the low range of libraries generally described for affinity maturation that is a range of mutation frequency of 0.4–0.8% [25], [26], 0,5–3% [27], 0.1–0.8% [28]. This evolved scFvC1 library had an estimated size of 8.10^6^ independent clones.

**Figure 1:**
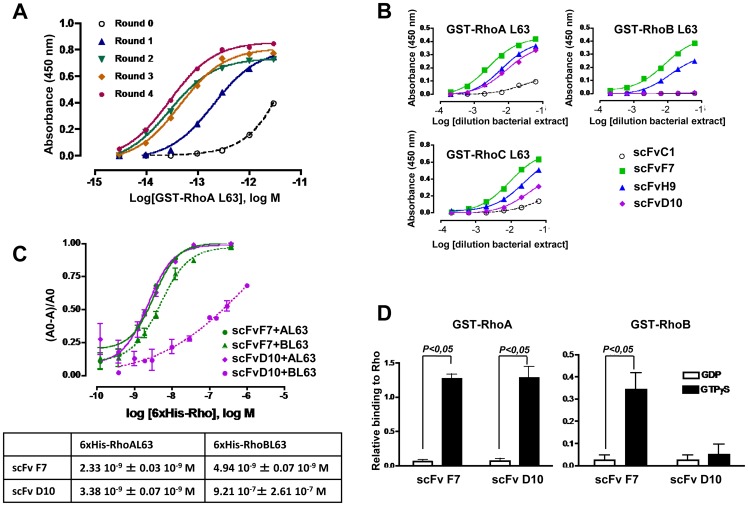
Affinity maturation revealed the possibility to obtain binders distinguishing RhoA and RhoC from RhoB active conformations. A, Improvement in apparent affinity throughout the rounds of selection was evaluated by a polyclonal phage ELISA on dilution of GST-RhoAL63 displayed in molar logarithmic scale (Log M). B, Three purified scFvs (F7, H9 and D10) were analyzed for their binding specificity towards L63 active mutants of recombinant GST-RhoA, RhoB and RhoC by ELISA. Purified scFvC1 was used as a control. C, Affinities of two scFvs (F7 and D10) for 6xHis-RhoAL63 (AL63), 6xHis-RhoBL63 (BL63) were measured by competitive ELISA as described in experimental procedures. K_d_ values were determined by nonlinear regression and listed in the insert table (mean ± SD, n = 3 each). D, The specificity of purified scFvF7 and scFvD10 for the active form of the recombinant wild type GST-RhoA and GST-RhoB loaded with either GDP or GTPγS were assessed by ELISA. Results are expressed as normalized absorbance of the scFvs to the total amount of coated GST-Rho quantified by the use of commercial antibodies (mean ± SD, Mann-Whitney test, n = 4 each).

In a first selection strategy aiming of affinity maturation, the library was panned by increasing the washing steps number and by lowering the antigen concentration during the successive rounds of selection. We used GST-RhoAL63 as bait which presents very low kinetics of GTP hydrolysis in order to preserve the active conformation of the protein all along the selection procedure. The first round of panning was carried out with saturating antigen coating in order to remove nonfunctional clones formed by the mutagenesis process and also allowing the amplification of rare and poorly expressed scFvs. To select binders with improved affinity, the second round was performed in a similar way but with an increased washing stringency. We further assessed by ELISA the antigen concentrations giving 70% and 30% of the maximal binding of phage outputs from the previous rounds (data not shown), then applied this decrease corresponding to 50 ng/mL and 5 ng/mL respectively to rounds three and four. Such decreased antigen concentrations, while keeping the input of phage constant, induced a competition between phages that display scFv very similar between each other [29]. We then compared the binding of the output phages obtained over the four selection rounds to GST-RhoL63 by polyclonal phage ELISA (Figure 1A). The results clearly indicated an increase of the signal, which can reflect an improvement of the affinity of the selected phage population. However, one can notice that the enrichment occurred as soon as the washing stringency was increased in the second round and that the effect of decreasing antigen concentrations appeared to be mild. At this stage, we cannot evaluate whether the antigen concentrations were not decreased enough to create sufficient competition or else the limit of the library was reached.

### Characterization of the selected single chain antibodies

Screening of individual clones was carried out by phage ELISA and 21 out of 94 clones were chosen for further analysis given their high signal in ELISA (data not shown). Among the selected clones, DNA sequencing revealed that two of them did not exhibit any mutation, therefore corresponding to the original scFvC1. The other clones presented amino acid substitutions mainly located in the CDR of the VH and VL domains sometimes associated with mutations located in the framework or the linker (data not shown). These data strongly suggested that the majority of the clones selected could have interesting properties as the CDR of the antibodies mainly determines their affinity and specificity properties [30]. Sequence analysis revealed that one variant possessing the N104S substitution located within the CDR3 VH was represented seven times while three others exhibited the same mutation with one or two others additional mutations. Furthermore, four clones harbored the R107G substitution located within the CDR3 VH with or without additional mutations. These observations were in favor of a specific enrichment during biopanning.

In a first set of experiment, assessment of apparent affinity of the selected clones was performed by clonal phage ELISA (data not shown) as well as in their soluble forms against a range of antigen concentrations *i.e.* the locked GTP mutant of RhoA, RhoB and RhoC (Figure 1B). As expected, their signal intensities were dramatically higher than the one of the scFvC1, suggesting an affinity improvement particularly for the clone F7. Two clones including D10 were unable to bind to RhoB activated form but were binding to RhoA and RhoC activated forms with high apparent affinity.

**Figure 2:**
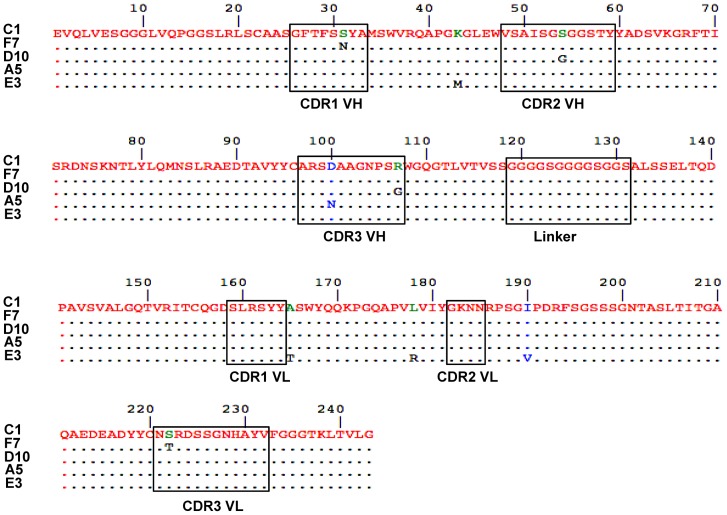
Protein sequence alignment of the characterized scFvs (F7, D10, A5, E3) showing the full sequence of scFvC1 and mutated aminoacid. Red to (-) correspond to aminoacid conservation. Blue amino acid correspond to mutation between strongly similar aminoacids. Green to black indicates aminoacid change between group of weakly conserved properties. Domains referred as complementary determining regions (CDR) of heavy chain (VH) and light chain (VL) are also indicated as well as the linker peptide between VH and VL.

We focused on F7 and D10 clones, which exhibited an apparent high affinity and different selectivities. ScFvF7 and scFvD10 were further analyzed in a competitive ELISA method that allow to assess the binding affinity [19], [20]. As shown in Figure 1C, binding affinities to RhoAL63 and RhoBL63 were similar for the scFvF7 (2.33.10^−9^ M and 4.94.10^−9^ M respectively), whereas scFvD10 recognized RhoBL63 with an IC_50_ value of only 9.21.10^−7^ M, corresponding of more than 200-fold decrease in affinity compared to that to RhoAL63 (3.38.10^−9^ M). Due to its very low affinity, scFvC1 could not be included in the above experiment. However, its affinity measured by Surface Plasmonic Resonance in a previous study (3.10^−6^ M) [16] permitted us to evaluate a gain of affinity of almost three orders of magnitude for scFvF7 and D10 towards RhoA. To ensure whether scFvF7 and D10 retain their respective selectivity towards the active form of the non mutated Rho, we next assayed their binding to RhoAwt and RhoBwt preloaded with GDP nucleotide or the slowly hydrolysable analogue of GTP, GTPγS. Results showed that the two scFvs selectively bound to recombinant RhoA-GTPγS, but that unlike scFvF7, scFvD10 failed to bind to the active form of wild type RhoB (Figure 1D). Aminoacid sequences of these two clones aligned to the scFvC1 are shown in Figure 2.

### Selection of an scFv against RhoB active conformation

**Figure 3:**
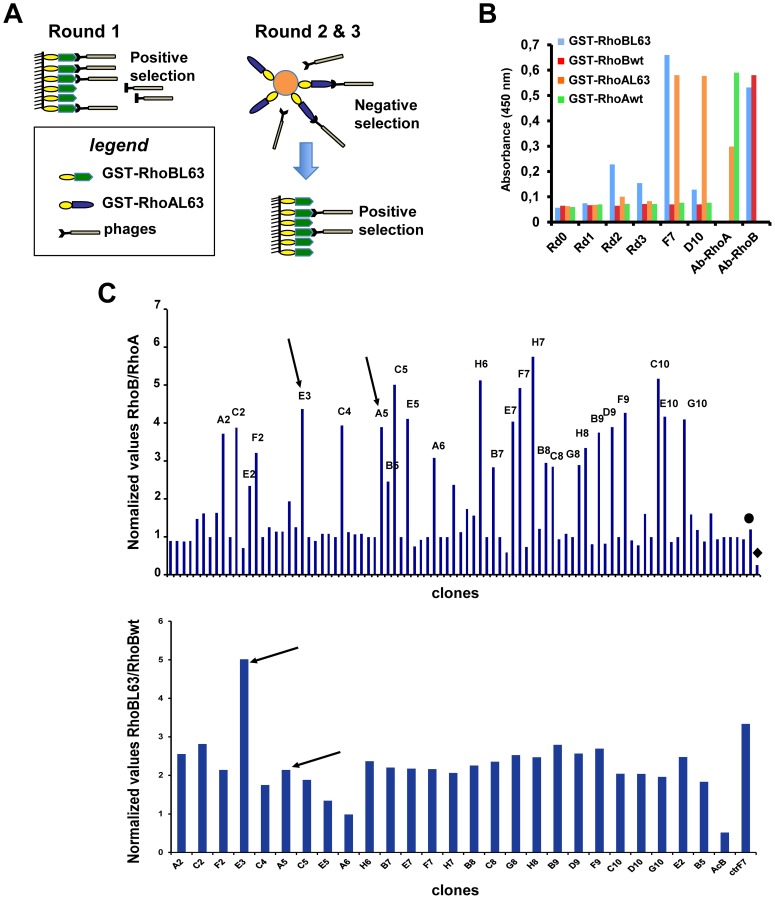
Selection of a RhoB active conformation specific scFv. A, Strategy of phage display selection. B, The enrichment of clones specific of the RhoB active form throughout the selection procedure was assessed by polyclonal phage ELISA on captured GST-Rho proteins from crude extract. Total amount of coated GST-Rho and active form of GST-RhoL63 were quantified with commercial anti-RhoA (Ab-RhoA) and anti-RhoB (Ab-RhoB) antibodies, and phageF7 (F7) and phageD10 (D10), respectively. GDP-bound GST-Rho (wt) was included as controls. C, (top panel) 88 individual clones were analyzed for their binding to GST-RhoBL63 and GST-RhoAL63 by phage ELISA. Results are expressed as the ratio of absorbance against GST-RhoBL63 *vs.* GST-RhoAL63. PhageF7 (black circle) and phageD10 (black diamond) were included as controls. Arrows indicate the clones E3 and A5 further selected. (Bottom panel) 26 clones were further analyzed for their binding to GST-RhoBL63 and GST-RhoBwt-GDP. Results are expressed as the ratio of absorbance against GST-RhoBL63 *vs.* GST-RhoBwt. Arrows indicate the clones E3 and A5. E3 was the best conformational sensor selective of active RhoB-GTP.

The results described above clearly demonstrated that the selection against active conformation of RhoA led to the isolation of evolved scFvC1 that can distinguish RhoA from RhoB active form. Therefore, we hypothesized that it could be possible to isolate from the scFvC1 library antibody fragments selective towards RhoB active conformation provided we used a suitable strategy of selection. To this aim, the scFvC1 library was first panned against GST-RhoBL63 with low washing stringency in order to amplify rare and poorly expressed scFvs. The two subsequent rounds of selection consisted in a counter-selection performed by pre-incubating the phages against GST-RhoAL63, thus removing scFvs able to bind to RhoA. The remaining unbound fraction was then panned against GST-RhoBL63 bound glutathione coated micro wells (Figure 3A) and amplified. Polyclonal ELISA using phages from each round of selection were showing an enrichment of phages able to selectively bind to RhoBL63 that was effective as soon as the second round (Figure 3B).

**Figure 4:**
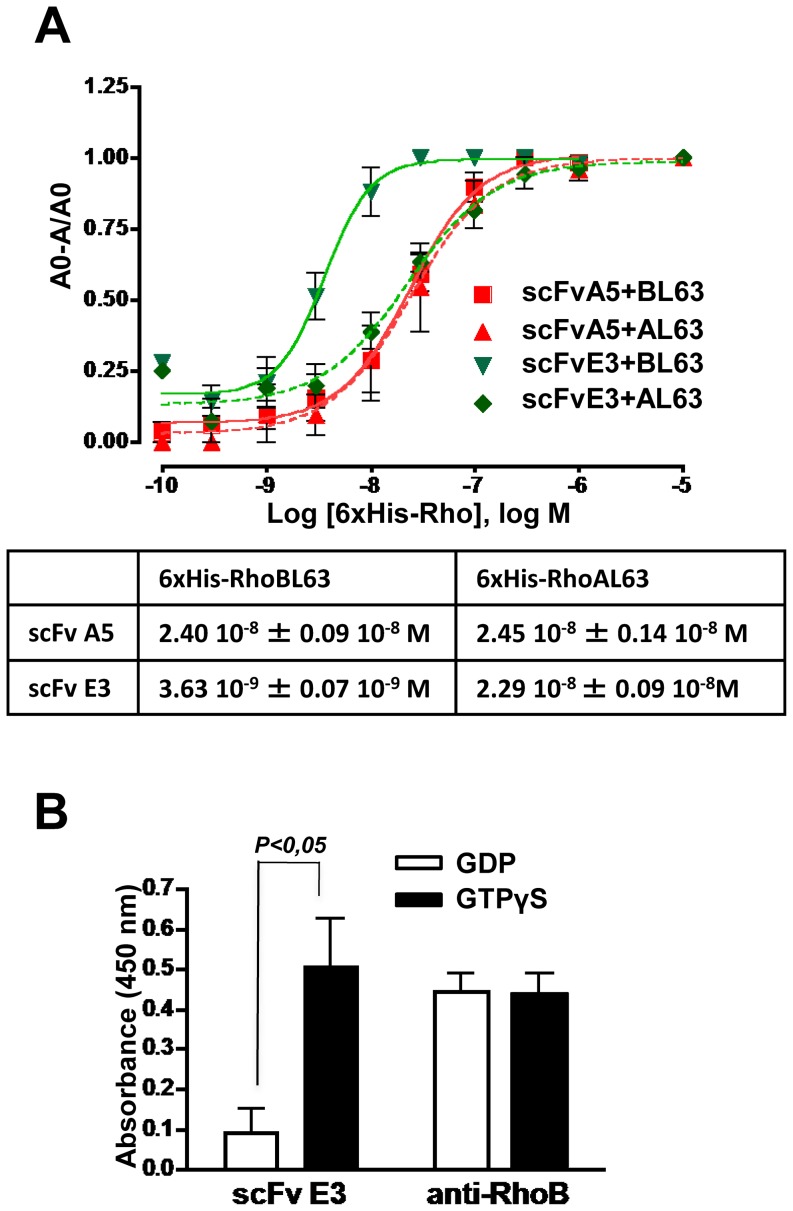
ScFvE3 preferentially binds to RhoB active conformation. A, Binding affinities of two selected scFvs (A5 and E3) for 6xHis-RhoAL63 (AL63), 6xHis-RhoBL63 (BL63) were measured by competitive ELISA on dilution of 6his-RhoL63 displayed in molar logarithmic scale (Log M), as described in experimental procedures. K_d_ values were determined by nonlinear regression and listed in the insert table (mean ± SD, n = 3 each). B, The specificity of the purified scFvE3 for the active form of the recombinant wild type GST-RhoB loaded with either GDP or GTPγS was assessed by ELISA. Results are expressed as absorbance at 450 nm (mean ± SD, Mann-Whitney test, n = 4).

Afterwards, 88 individual clones were examined for their selective binding to RhoBL63 with respect to RhoAL63. 26 phages exhibiting a ratio greater than or equal to 3 were further analyzed for their capability to selective bind to RhoBL63 with respect to RhoBwt that is mainly in the GDP form (Figure 3C). DNA sequencing revealed that the D100N substitution located within the CDR3 VH with or without additional mutations was found in all but one selected clones. Furthermore, one clone having the unique D100N substitution was represented five times while two others clones were found identical. Only the scFvE3 differed from the others clones as it had 4 substitutions located within the scaffold of the antibody fragment (Figure 2). We first measured the differential affinity to RhoBL63 *vs.* RhoAL63 of the scFvA5 displaying the unique D100N substitution and the scFvE3 devoid of this mutation by performing a competitive ELISA. Surprisingly, only the scFvE3 displayed a higher affinity towards RhoB (3.63 10^−9^ M) than RhoA (2.29 10^−8^ M), corresponding to a differential affinity factor of 6 in this assay (Figure 4A). At this stage, no explanation could be advanced concerning the fact that the D100N substitution shared by the majority of the selected clones did not result in an increase of affinity towards RhoB. We further focused on characterization of the scFvE3. Finally we confirmed its selectivity for the active form of RhoB by comparing its binding with non mutated RhoB loaded with nucleotides *i.e.* GDP or GTPγS (Figure 4B).

### Kinetic Affinity measurements of selected scFvs

**Figure 5:**
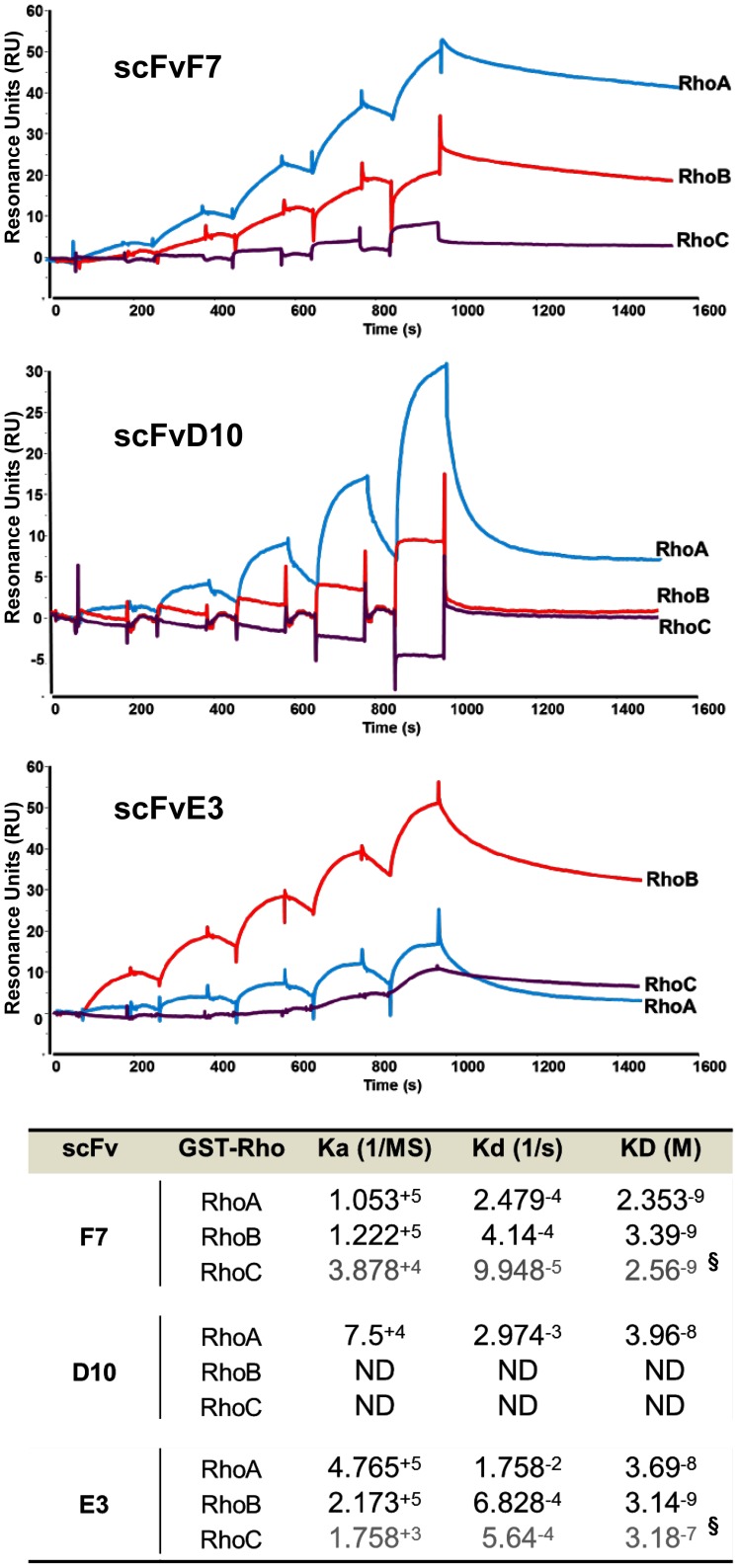
Real-time binding of scFvs F7, D10 and E3 by Surface Plasmon Resonance on immobilized GST fusion Rho active mutant proteins. Single Cycle Kinetics analysis was performed on immobilized GST fusion proteins RhoAL63, RhoBL63 and RhoCL63 (1000 RU) with five injections of analyte at 6.25nM, 12.5nM, 25nM, 50nM, and 100nM. Analyte injections lasted for 120 s each and were separated by 180 s dissociation phases. An extended dissociation period of 10 min followed the last injection. The two sensograms recorded for a given analyte were fitted globally to a 1∶1 interaction (data not show). Each sensogram represents a differential response where reference channel of immobilized GST protein has been substracted and is expressed in RU as a function of time in second. Bottom: table summarizing kinetic constant parameters. ND means not determined. § means that kinetics parameters were obtained by fitting curves with too low resonance units to give accurate values.

Molecular evolution led us to discover scFvs showing apparent high affinity towards the 3 Rho or more selectivity between RhoA/C and RhoB. To get insight into the interaction properties of scFvF7, D10 or E3 we performed real-time binding measurements by Surface Plasmonic Resonance (SPR). We confirmed affinities and selectivities measured by ELISA for the scFvs F7 and E3 towards RhoA and RhoB active mutants. Whereas scFvF7 harbored almost the same K_d_ values in the nanomolar range toward these 2 antigens, affinity of scFvE3 appeared to be even 10 times higher towards RhoB than RhoA mainly due to a faster dissociation rate on RhoA protein (Figure 5). However affinity measurements towards GST-RhoCL63 seemed to be unreliable as the number of resonance units was too low to give an accurate determination of the kinetic parameters. Finally, SPR affinity measurements of the scFvD10 were impeded by the poor stability of this peculiar scFv as resonance units signal were always low and K_d_ values could not even be determined for RhoC. The affinity towards RhoA was found ten times lower than the one obtained with ELISA. Nevertheless, a selectivity of the scFvD10 for RhoA vs RhoB was confirmed as no binding towards RhoB was observed.

### Pulldown of endogenous RhoB activity

**Figure 6:**
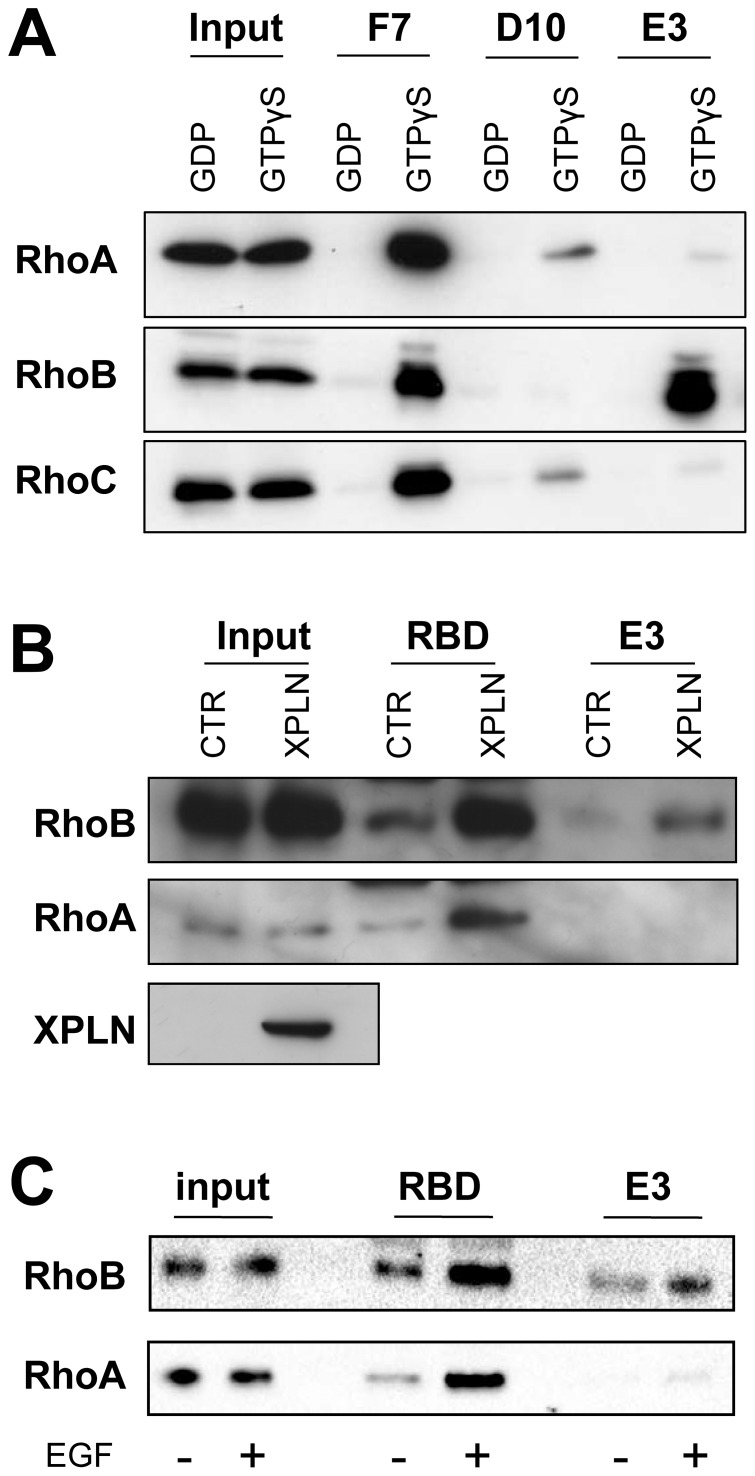
scFvE3 is a selective sensor of RhoB activation in HeLa cells. A, CBD-pulldown experiments on nucleotides loaded HeLa cell extracts showing the specificities of the selected scFvs. HeLa cell extracts were loaded with either GDP (1 mM) or GTPγS (100 µM) and incubated with scFvs F7, D10 and E3 fixed on chitin beads. CBD-pulldowns were analyzed by Western blotting using anti-RhoA, anti-RhoB and anti-RhoC antibodies. Total extract used for CBD-pulldown is indicated as *input* and examined by western blotting with the same antibodies. Western Blot is representative of 4 independent experiments. B, RhoB and RhoA activation were assessed by GST-pulldown (RBD) and CBD-pulldown (E3) experiments with cell lysate from HeLa cells transiently transfected with plasmids expressing Myc-tagged XPLN or GFP under the control of CMV promotor. XPLN was detected by using an anti-c-myc antibody. C, HeLa cells were serum-starved for 24 h and treated with EGF (2.5 ng/mL) for 10 min before lysis then RhoB and RhoA activation were assessed by GST-pulldown (RBD) and CBD-pulldown (E3) experiments. Beads-bound proteins were analyzed by Western blotting using anti-RhoA and anti-RhoB antibodies. Total cell extracts are indicated as *input* and examined by western blotting with the same antibodies. Western Blots are representatives of 2 independent experiments.

Rho proteins are subjected to multiple post-translational modifications such as prenylation [31], [32], palmitoylation [33] as well as phophorylation [34], [35]. Given that the selection of scFvs was carried out against bacterially expressed recombinant antigens devoid of any known post-translational modifications, there was a need to confirm that the selected scFvs were able to recognize active Rho derived from eukaryotic cells. To this end, scFvs were used in active Rho pull down experiment similarly as the conventional Rho-binding domain of Rhotekin (RBD) in GST pulldown. As GST fusion of scFvs were unstable in the secretion pathway, we first expressed them in fusion with the N-terminal of the Chitin Binding Domain (CBD) of the chitinase A1 which has been shown to bind with high affinity to chitin [36] and were purified onto chitin-coated beads. In order to artificially control the amount of antigen in the inactive or active conformation, HeLa cells extracts were first loaded either with GDP or GTPγS, then mixed with scFvs-bound beads referred as CBD-pulldown. Rho proteins specifically bound to the scFvs-beads were analysed by Western Blot with commercial anti-RhoA, anti-RhoB and anti-RhoC antibodies. The scFvF7, D10 and E3 interacted specifically with the GTPγS-loaded Rho proteins. Moreover, the selectivity that we observed on recombinant Rho proteins were fully retained on endogenous active form of each Rho, namely that the scFvF7 recognized RhoA, RhoB and RhoC, the scFvD10 RhoA and RhoC, the scFvE3 only RhoB (Figure 6A).

We next focused on the ability of scFvE3 to specifically bind to endogenous activated RhoB in the cellular complexity. This was first assessed by CBD-pulldown on HeLa cells transiently expressing the Myc-tagged XPLN that stimulates guanine nucleotide exchange on RhoA and RhoB, but not RhoC [37]. As a control, we performed an RBD-pulldown to specifically precipitate GTP-bound RhoB and RhoA from cell extracts. As shown in Figure 6B, the scFvE3 only interacted with GTP-bound RhoB whereas RhoA was also activated as revealed by the RBD interaction. These results were confirmed when HeLa cultured cells were stimulated with Epidermal growth factor (EGF) which has been shown to transitory activate RhoA and RhoB GTPases [21] (Figure 6C).

## Discussion

Phage display technology has been proved to be effective in raising antibodies with markedly enhanced specificities as well as in improving antibodies binding affinities. We performed an affinity maturation by introducing random mutations within the scFvC1 coding DNA that recognizes GTP-bound form of RhoA, RhoB and RhoC. Affinity maturation can be conducted by a strategy based on the off-rate selection [25], [38] or a competition between the binders [25], [28], [39], [40]. As we wanted to preserve the active conformation of Rho proteins all along the selection procedure, we chose an affinity selection to avoid long incubation time required in off-rate selection protocols [24]. This approach was successful since we obtained scFvs with affinities dramatically higher than the one of the scFvC1, while keeping the properties of being a conformational sensor.

The phylogenetic tree of small GTPases classifies into subfamilies among which the RhoA, RhoB and RhoC members shares a high homology in terms of secondary structure [5]. This homology assessed to more than 80% identity on the whole protein reaches more than 95% in the 100 amino-terminal residues that comprises the nucleotide binding loop and the switch I and II which are supposed to be implicated in the conformational recognition by effector proteins [3]. Despite this fact, molecular evolution of the scFvC1 permitted us to isolate scFvs able not only to discriminate Rho proteins in their active conformation but in addition to selectively bind to either RhoA/C or to RhoB. Strikingly, the differential of affinity of the scFvE3 for RhoB versus RhoA active conformation is quite modest (10 fold as determined by SPR experiments) but appeared to be sufficient to pull down specifically an activation of RhoB in cells stimulated by EGF. Rare studies have reported the isolation of conformation specific antibodies discriminating active conformations of small GTPases using phage display technology such as HRas [41], Rab6A [42], Rho [16], and our results confirm the efficiency of this fully in vitro strategy. Furthermore, as previously reported by Tanaka and Rabbitts this approach can constitute the first step in order to achieve the isolation of intrabodies when coupled to intracellular antibody capture technique [43].

**Figure 7:**
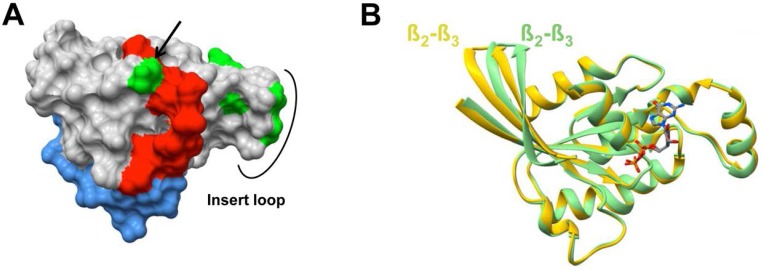
Structure comparison between RhoA and RhoB. A, The filling structure of active RhoAV14-GTPγS (Pdb code 1A2B) was created by UCSF-Chimera software [53]. Switch I & II are depicted in red and blue, respectively. The RhoA/RhoB differences in amino acids sequence are shown in green with the residue 29 indicated by an arrow. B, Structural divergence between RhoA and RhoB in the β2-β3 région. The *Matchmaker tool* in *UCSF-Chimera* (http://www.cgl.ucsf.edu/chimera) was used to generate the structural superposition. RhoA (Green, Pdb code 1DPF) & RhoB (Yellow, Pdb code 2FV8) structures are shown in ribbon model and the substrate GDP in stick. Both proteins are in the inactive form with only GDP in the active site.

To date we do not know the exact epitopes where these scFvs bind on Rho proteins and the mechanism that allow scFvE3 to discriminate RhoB from RhoA remains unknown without performing co-crystallization studies. Nevertheless we assume that these conformational sensors may interact with residues near the switch regions as it has been shown for effector proteins *e.g.* mDia1 [44], PKN/PRK1 [45], ROCKI [46] and in a remarkable way for an anti-Hras-GTP intrabody [47]. Interestingly, this region is extremely homologous in secondary structure alignment between RhoA and RhoB, apart from the residues 10 and 29. Modeling these residue discripencies onto the filling structure of RhoA-GTP, since the GTP-bound form structure of RhoB has not been resolved until now, reveals that only position 29 in the switch I is exposed indeed (Figure 7A). Moreover 4 residues within the insert loop are as well exposed and might also explain the differential binding of the scFvs (Figure 7A). Apart from the switch domains, the ß2/ß3 region has been described to be involved in the specific binding of RhoA effectors [45], [48]. As this region positioning does not change whatever the nucleotide bound in RhoA resolved structures, we superimposed available ribbon models of RhoA and RhoB inactive conformations. Strikingly, we observed a clear shift in this ß2/ß3 region at the protein surface (Figure 7B), that could be involved in the scFvs selectivities. The knowledge of the exact residues really implicated in the scFv binding would be of great importance to optimize affinity and selectivity of scFv by a targeted approach. This will be reached by co-crystalisation of Rho in the presence of their selective scFv. Nevertheless, we cannot exclude that the carboxy-terminal domain could be part of the binding site as it has been shown for the effector proteins selective of RhoB, MAP1A/LC2 [49] and p76^RBE^ [50].

Rho GTPases become activated among several stimuli, then trigger signaling pathways that control many cellular processes, the deregulation of which may lead to disease such as cancer. The intracellular level of Rho GTP-bound form represents a criteria of choice to characterize the activity of these pathways and to understand physiopathological processes. To date, we use the ability of the Rho binding domain of Rhotekin to selectively bind to RhoA, RhoB, RhoC GTP-bound form to discriminate the ratio between the GTP and GDP-bound form of Rho Proteins in cellular conditions in a semi-quantitative manner. This technique implies that activated Rho bind the RBD with the same affinity, which is in the range of 100nM [51]. However, the RBD recombinant domain is poorly stable and does not tolerate many tags or expression systems, remaining expressed as a GST fusion tool. Its relatively low affinity combined to its labile stability implies that the assay has to be done in critical scale condition and cannot be engineered to perform precise quantitation of Rho cellular activation [52]. Nevertheless, our results showed that scFvs could recognize with higher affinity than the RBD and with at least similar selectivity the GTP-bound Rho, some scFv being even more selective to a single Rho. Therefore our study opens up all the potential of scFv engineering tools to implement other recombinant format, with different tags or multimeric status, which will allow the establishment of reliable quantitation biosensors to address Rho activity biological function in vitro as well as in the cellular context [43]. Actually, deciphering intracellular signaling pathways requires sophisticated tools able to monitor not only the expression of proteins participating in the signal transduction but also the status of activation of switch proteins, witnesses of the implication of a particular pathway in a physiological phenomenon.
